# Rotavirus G9P[4] in 3 Countries in Latin America, 2009–2010

**DOI:** 10.3201/eid1908.130288

**Published:** 2013-08

**Authors:** Osbourne Quaye, Sharla McDonald, Mathew D. Esona, Freda C. Lyde, Slavica Mijatovic-Rustempasic, Sunando Roy, Dina J. Castro Banegas, Yolanda Mencos Quiñonez, Blanca L. Chinchilla, Fabián Gómez Santiago, Herlinda García Lozano, Gloria Rey-Benito, Lúcia H. de Oliveira, Jon R. Gentsch, Michael D. Bowen

**Affiliations:** Centers for Disease Control and Prevention, Atlanta, Georgia, USA (O. Quaye, S. McDonald, M.D. Esona, F.C. Lyde, S. Mijatovic-Rustempasic, S. Roy, J.R. Gentsch, M.D. Bowen);; Nacional Colonia La Campaña, Tegucigalpa, Honduras (D. J. Castro Banegas);; Ministerio de Salud Pública y Asistencia Social, Guatemala City, Guatemala (Y. Mencos Quiñonez, B.L. Chinchilla);; Instituto de Diagnóstico y Referencia Epidemiológicos, Mexico City, Mexico (F. Gómez Santiago, H. García Lozano);; Pan American Health Organization, Washington, DC, USA (G. Rey-Benito, L.H. de Oliveira)

**Keywords:** rotavirus, rotavirus G9P[4], viruses, VP7, VP4, NSP4, reassortant, Latin America, Mexico, Guatemala, Honduras, enteric infections

**To the Editor:** Group A rotaviruses are the most common viral cause of acute gastroenteritis in young children. The most frequently detected group A rotavirus genotype combinations include G1P[8], G2P[4], G3P[8], G4P[8], and G9P[8]. The G9 genotype has been associated with multiple P types, including P[8], P[6], and P[4], although genotype G9P[8] is predominant (*1*).

In Latin America, a large number of unusual G-P combinations have been reported, and among these is the rare G9P[4] genotype, which was identified in Brazil in the 1990s (*2*), and later reported infrequently elsewhere in Latin America (*3*). In 2010, cases of group A rotavirus gastroenteritis associated with genotype G9P[4] were reported in Mexico (*4*). Increases in the incidence of group A rotavirus gastroenteritis were reported in 2010 in Mexico and Guatemala and in 2009 in Honduras (http://new.paho.org/hq/dmdocuments/2010/Epi_Alerts_2010_mar_5_rotavirus.pdf).

In response to these reports of increased group A rotavirus disease, fecal samples collected in Chiapas State, Mexico (in 2010, 30% of the cases in Mexico were from Chiapas), Guatemala, and Honduras in 2009–2010 that were positive by enzyme immunoassay were sent to the US Centers for Disease Control and Prevention (Atlanta, GA, USA) for characterization. Viral protein 4 (VP4) (P) and VP7 (G) genotyping, nucleotide sequencing, and genotype identification were performed by using consensus and genotype-specific oligonucleotide primers (*5*), and sequences were subjected to phylogenetic analyses. VP6 and nonstructural protein 4 (NSP4) genes of selected samples were also sequenced.

For 26 samples from Mexico, G9 accounted for ≈90% of all the G types; all samples had mixed P types. Approximately 80% of samples were genotype G9P[4,8]; genotypes G3P[4,8], G3,9P[4,8], and G9P[4,9] accounted for the remaining samples. We hypothesize that the G9P[4,8] genotype was the result of mixed G9P[4] and G9P[8] infections by strains with homologous G9 VP7 genes. For 41 samples from Guatemala, G9P[4] accounted for 66%, followed by G9P[8] (32%), and G3,9P[4,8] (2%). For 50 samples from Honduras, 50% were G1P[8] and 36% were G9P[4]. G3P[8], G1,3P[8], and G4P[6] comprised the remaining samples.

Results showed an increase in prevalence of the rare G9P[4] strain, which was the predominant strain in Guatemala and Mexico, and the second most predominant strain in Honduras, after G1P[8]. Group A rotavirus genotypes G1P[8] and G3P[8] have been reported to be predominant in Mexico (*4*). G1P[8] and G2P[4] were associated with most group A rotavirus infections in Guatemala (*6**,**7*) and G2P[4] predominated in Honduras (*7*). G9P[4] has not been reported in Guatemala or Honduras. In Mexico, the outbreak might have originated from a common source, such as untreated drinking water (http://new.paho.org/hq/dmdocuments/2010/Epi_Alerts_2010_mar_5_rotavirus.pdf).

Phylogenetic analysis of G9 gene sequences from the 3 countries showed that they clustered in a sublineage and were closest to G9P[8] strains circulating globally (Figure, Appendix, panel A). VP4 genes from the 3 countries also clustered within a sublineage of a clade containing global strains (Figure, Appendix, panel B). VP6 sequences clustered within a sublineage of the I2 genotype clade (Figure, Appendix, panel C). NSP4 gene sequences clustered within a sublineage of the E6 genotype clade, which they shared with group A rotavirus strains from India and Bangladesh (Figure, Appendix, panel D).

**Figure:**
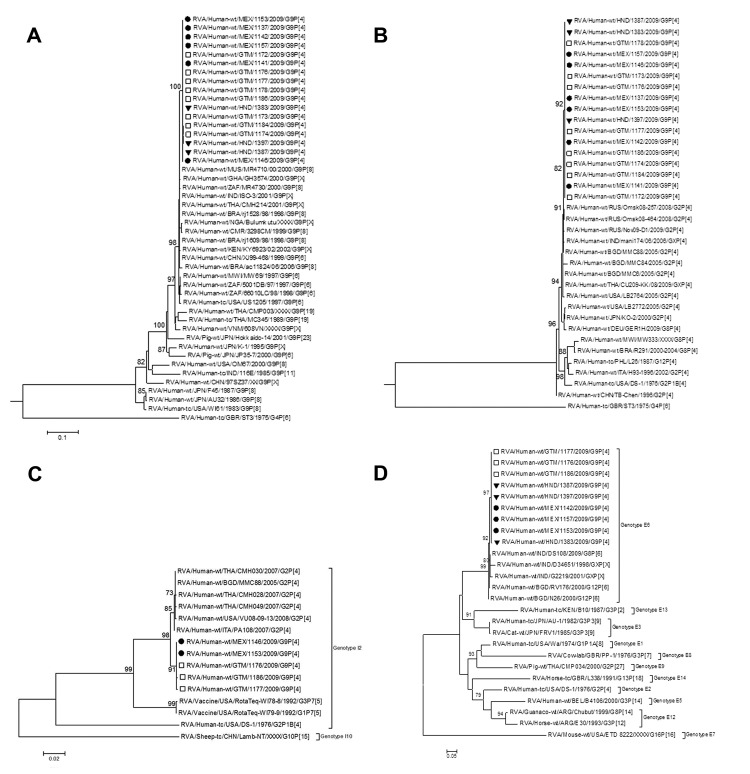
Maximum-likelihood phylograms indicating genetic relationships of nucleotide sequences of A) viral protein 7 (VP7), B) VP4, C) VP6, and D) nonstructural protein 4 (NSP4) genes of human G9P[4] group A rotavirus (RVA) strains from Mexico, Guatemala, and Honduras, and sequences of human and animal RVA strains from GenBank. Partial VP4 (VP8* region), VP7 and VP6 gene sequences (742, 783, and 1,155 bases, respectively) and complete gene sequences of NSP4 (528 bases) were aligned with cognate reference strain sequences by using ClustalW in MEGA 5.05 (http://megasoftware.net/mega.php). The optimal evolutionary model that best fit each sequence dataset was identified by using MEGA 5.05. Maximum-likelihood trees were constructed by using SEAVIEW version 4 (www.seaviewfishing.com/DownloadSoftware.html), and approximate likelihood ratio test (aLRT) statistics were computed for estimation of branch support. On the basis of Akaike information criteria with a correction for finite sample sizes, we selected the Tamura-Nei plus gamma, general time reversible plus gamma, general time reversible plus invariant sites, and Hasegawa-Kishino-Yano plus gamma models for genes VP4, VP7, VP6, and NSP4, respectively. Trees are drawn to scale. Only aLRT values ≥70% are shown. Solid circles indicate G9P[4] strains from Mexico, squares indicate G9P[4] strains from Guatemala, and solid inverted triangles indicate G9P[4] strains from Honduras sequenced in this study. Scale bars indicate genetic distances. MEX, Mexico; GTM, Guatemala; HND, Honduras; MUS, Mauritius; GHA, Ghana; ZAF, South Africa; IND, India; THA, Thailand; BRA, Brazil; NGA, Nigeria; CMR, Cameroon; KEN, Kenya; CHN, China; MWI, Malawi; USA, United States; VNM, Vietnam; JPN, Japan; GBR, United Kingdom; RUS, Russia; BGD, Bangladesh; DEU, Germany; PHL, The Philippines; ITA, Italy; BEL, Belgium; ARG, Argentina.

The high degree of genetic similarity among these strains in all 4 genes (99.6%–100%), as demonstrated in this study, suggests that strains from all 3 countries had a common origin. In regions of overlapping sequence, VP4 gene sequences from this study shared 98.3%–100% identity (408 bases) with G9P[4] strains from Mexico (GenBank accession nos. JN180414–JN180451), and VP7 gene sequences shared 97.9%–98.9% identity (97 bases) (GenBank accession nos. JN180376–JN180413).

Rahman et al. have hypothesized that the G9P[4] genotype combination was formed by reassortment between more frequently occurring strains (e.g., G2P[4] and G9P[6] strains) (*8*). Potential parental strains have been circulating at high levels in Latin America for ≈30 years. During this period, G9 and P[4] accounted for 15% and 22% of all G and P types, respectively, in Latin America and the Caribbean (*3*). Only 0.4% of strains were G9P[4] during this period, which suggests that the markedly increased prevalence of this genotype in 2009–2010 was the result of a dramatic event, such as genetic reassortment.

Previous studies of G9P[4] strains examined only VP4 and VP7 genes and had not characterized VP6 and NSP4 genes of these strains. The presence of an NSP4 genotype E6 gene within these viruses was surprising. The NSP4 E6 genotype has been described in only 5 strains, all of which were from human cases of infection in Bangladesh or India (*9**,**10*) and were associated with VP4 genotype P[6] and VP7 genotypes G8 or G12. The complete global distribution of this NSP4 genotype remains to be determined.

Although many factors account for increased reports of group A rotavirus gastroenteritis observed in Mexico, Guatemala, and Honduras in 2009–2010, our data suggest emergence of the previously rare G9P[4] group A rotavirus genotype in these countries. Whether the G9P[4] genotype becomes the common strain in Latin America or elsewhere remains to be determined.
